# Vitamin D Levels in COVID-19 Outpatients from Western Mexico: Clinical Correlation and Effect of Its Supplementation

**DOI:** 10.3390/jcm10112378

**Published:** 2021-05-28

**Authors:** Gabriela Athziri Sánchez-Zuno, Guillermo González-Estevez, Mónica Guadalupe Matuz-Flores, Gabriela Macedo-Ojeda, Jorge Hernández-Bello, Jesús Carlos Mora-Mora, Edsaúl Emilio Pérez-Guerrero, Mariel García-Chagollán, Natali Vega-Magaña, Francisco Javier Turrubiates-Hernández, Andrea Carolina Machado-Sulbaran, José Francisco Muñoz-Valle

**Affiliations:** 1 Institute of Research in Biomedical Sciences, Department of Medical Clinics, University Center of Health Sciences (CUCS), Edificio Q, 950 Sierra Mojada, Guadalajara 44340, Mexico; athziri_93_7@hotmail.com (G.A.S.-Z.); guillermo.gonzalezestevez@cucs.udg.mx (G.G.-E.); matuzmonica@gmail.com (M.G.M.-F.); gaby_macedo@yahoo.com.mx (G.M.-O.); jorge89_5@hotmail.com (J.H.-B.); edsaul.perez@gmail.com (E.E.P.-G.); maye_999@hotmail.com (M.G.-C.); alejandra.vega@academicos.udg.mx (N.V.-M.); ln.paco.turrubiates@gmail.com (F.J.T.-H.); 2 COVID-19 Situation Room (Analysis Group), University of Guadalajara, Guadalajara 44340, Mexico; 3 Laboratory for the Diagnosis of Emerging and Reemerging Diseases (LaDEER), University Center for Health Sciences (CUCS), Edificio Q, 950 Sierra Mojada, Guadalajara 44340, Mexico; jesus.moram@academicos.udg.mx; 4 Institute for Research in Childhood and Adolescent Cancer (INICIA), Department of Human Reproduction, Child Growth and Development Clinics, University Center for Health Sciences (CUCS), Edificio Q, 950 Sierra Mojada, Guadalajara 44340, Mexico; andrecaroms@gmail.com

**Keywords:** vitamin D, ergocalciferol, cholecalciferol, coronavirus, COVID-19, SARS-CoV-2, Latin America, biochemical parameters, supplementation

## Abstract

Background: The immunomodulatory effects of vitamin D are known to be beneficial in viral infections; it is also known that its deficiency is associated with a prognosis more critical of Coronavirus Disease 2019. This study aimed to determine baseline vitamin D serum concentrations and the effects of its supplementation in asymptomatic or mildly symptomatic Coronavirus Disease 2019 outpatients. Methods: 42 outpatients were included, 22 of which received a supplement of 10,000 IU of vitamin D3 for 14 days; the remaining 20 outpatients were designated as a control group. Serum levels of transferrin, ferritin, vitamin D, and D-dimer were measured at baseline in both groups. After 14 days, serum levels of total vitamin D were determined in the supplemented group. Results: At baseline, only 19% of infected outpatients had vitamin D levels corresponding to sufficiency. All outpatients with vitamin D insufficiency had at least one symptom associated with the disease, while only 75% of patients with symptoms presented sufficiency. On the seventh and fourteenth day of follow-up, the supplemented group presented fewer symptoms with respect to those non-supplemented. A vitamin D3 dose of 10,000 IU/daily for 14 days was sufficient to raise vitamin D serum concentrations. Conclusions: Immunomodulatory effects of vitamin D appear to be linked to the development of symptoms in positive outpatients. Vitamin D supplementation could have significant benefits in the Western Mexican population.

## 1. Introduction

Coronavirus Disease 2019 (COVID-19) is caused by the Severe Acute Respiratory Syndrome of Coronavirus 2 (SARS-CoV-2) [1]. According to the World Health Organization (WHO), as of January 2021, the number of confirmed cases in America was 36,943,389; meanwhile, in Mexico, official reports confirmed a total of 1,448,755 cases with 127,213 deaths [2]. As reported by Johns Hopkins University, these figures make Mexico the country with the highest observed case-fatality ratio [3].

Concerning SARS-CoV-2 infection, it is known that the virus has a tropism for the upper and lower respiratory tracts. Once it infects and starts replicating, the course of the disease can be mild or moderate; in some cases, it can even progress to severe pneumonia and Acute Respiratory Distress Syndrome (ARDS) [4]. The hallmark of COVID-19 pathogenesis is the excessive activity of immune cells, such as macrophages and T helper 1 cells (Th1), with the consequent release of pro-inflammatory cytokines, including interferon-gamma (IFN-γ), tumor necrosis factor-alpha (TNF-α), interleukin (IL)-1β, IL-6, and IL-8, that exacerbate the inflammatory response and mediate alveolar and endothelial damage [4,5].

Due to the importance of early diagnosis of this disease, a series of biochemical parameters have been identified as possible biomarkers of its severity [6], including lymphopenia and increased D-dimer levels ferritin troponin and C-reactive protein (CRP). These biomarkers are directly correlated with the coagulation, metabolism, hepatic, and renal function alterations in COVID-19 [7]. In addition, the AB0 blood group [8,9], older age, and specific comorbidities (such as cardiovascular diseases, hypertension, chronic respiratory disease, diabetes, chronic kidney disease, asthma, and obesity) are other factors associated with greater susceptibility or a worse prognosis of COVID-19 [10,11,12].

In the early stages of the disease, the protective immune response is responsible for successfully clearing the virus and resolving the infection. Hence, it is imperative to concentrate efforts on searching for prophylactic strategies that can improve this immune response [4]. As reported by Turrubiates et al., vitamin D intervention as an adjunctive treatment may be crucial in severe cases of COVID-19 with low 25-hydroxyvitamin D [25(OH)D] levels. Similarly, supplementation in therapeutic doses may help prevent SARS-CoV-2 infection, since the active metabolite of vitamin D exerts biological activities in the innate immune system [13,14].

Several studies have established that vitamin D can reduce the risk of infections and deaths from COVID-19 through different mechanisms: the maintenance of cell junctions [15] through the down-regulation of an Angiotensin-Converting Enzyme 2 (ACE2) receptor in host lung epithelial cells [16] and diminishing the cytokine storm by decreasing the expression and secretion of IFN-γ and pro-inflammatory cytokines, such as TNF-α and IL-6, as well as reducing chemokines, such as CXCL8 and CXCL10, [17] and modulating adaptive immunity by suppressing Th1 lymphocyte responses, in addition to promoting Treg lymphocyte polarization [18]. In addition to the above, recent studies have reported the association of vitamin D deficiency status with symptoms such as fever in COVID-19 positive patients [19].

Contrary to the growing number of publications pointing to the beneficial effects of vitamin D in patients with severe COVID-19 [20,21,22,23], studies are limited only to the patients with mild symptoms in patients with mild symptoms.

Despite the multiple beneficial effects of vitamin D supplementation, vitamin D deficiency is a common disorder (<10 ng/mL is considered an indication of vitamin D deficiency, according to suggested laboratory reference values) [24]. Consequently, an increase in the intake of foods rich in vitamin D and its supplementation has been recommended by different organisms for healthy and sick individuals to normalize their serum concentrations. Previous studies have recommended a vitamin D3 supplementation of 10,000 IU/day for a few weeks to increase serum levels of 25(OH)D in patients with low circulating levels (below 50 nmol/L). It has also been reported that doses of up to 15,000 IU daily of vitamin D are safe and effective in rapidly increasing 25(OH)D concentrations, followed by a maintenance dose of up to 5000 IU/day [5,25].

This study aimed to determine the baseline vitamin D serum concentrations in asymptomatic or mildly symptomatic COVID-19 outpatients and evaluate the effects of supplementation with 10,000 IU/daily of vitamin D3 and its relationship with biochemical parameters and clinical features.

## 2. Materials and Methods

### 2.1. Study Population

This study was designed as a randomized clinical trial conducted in 42 COVID-19 outpatients of both genders, diagnosed by the real-time PCR (qPCR) method using the DeCoV19 kit for qPCR and the reverse transcription (RT qPCR) Triplex Kit (Genes2life Cat. G2L-DeCoV19-MP. Guanajuato, Mexico), capable of detecting the N1, N2, and N3 genes of SARS-CoV-2, as specified by the manufacturer. The patients were recruited from the Laboratorio de Diagnóstico de Enfermedades Emergentes y Reemergentes (LaDEER) from the Universidad de Guadalajara.

The inclusion criteria for the present analysis were individuals with mild disease, over 18 years of age, who were not taking any vitamin D supplementation at the recruiting time. Informed consent was obtained from all patients enrolled in this study.

Patients were then randomized into two groups: the intervention group was formed by 22 patients who received oral supplementation of 10,000 IU daily of vitamin D3 in soft capsule form for 14 days. The intake was prescribed in the morning with the company of a meal. The remaining 20 patients that did not receive supplementation were designated as a control group. The study was conducted based on the Declaration of Helsinki rules and approved by the Institutional Ethics Committee of the Universidad de Guadalajara, Faculty of Medicine (folio number: CI-07620).

### 2.2. Clinical Assessment

For each patient, follow-ups were carried out from the day of COVID-19 diagnosis and continued for 14 days. Demographic data and past clinical history (including the presence of comorbidities, such as hypertension, diabetes, ischemic heart disease, asthma, and immunocompromised conditions) were recorded. Data such as blood groups were also considered for the analysis. In both study groups, a survey was applied to record the following symptoms: fever, cough, sore throat, dyspnea, chills, headache, myalgia, and gastrointestinal symptoms or if they were asymptomatic. Vital signs (pulse rate, blood pressure, respiratory rate, body temperature, and oxygen saturation on room) were also recorded for each patient (Supplementary Material). The calculation of the National Early Warning Score (NEWS) was also performed using a web calculator to determine the degree of illness of a patient. A score ≥ 5 was considered to remit the patients to medical review to possible clinical intervention and more intensive monitoring [26].

A structured survey was performed to identify the pharmacological treatment in the outpatients at baseline and follow-up. Treatment associated with COVID-19 was considered if patients were treated with any of the following drugs: analgesics, antipyretics, anticoagulants, antibiotics (azithromycin, erythromycin), antihistamines, beta-blockers, corticoids, anti-flu, and antiparasitic (ivermectin).

### 2.3. Laboratory Determinations

Serum samples were collected in both groups to measure baseline concentration of total 25(OH)D, D-dimer, and ferritin by chemiluminescence, as well as transferrin by turbidimetry. After 14 days, serum levels of 25(OH)D were also determined in the intervention group. Patients were classified as patients with vitamin D deficiency if they presented serum levels <20 ng/mL or insufficiency with levels of 20–29.9 ng/mL, while levels ≥30 ng/mL were considered as sufficiency [27].

In addition, a qualitative determination of antibodies against COVID-19 in blood was performed by the lateral flow immunoassay method (rapid test Certum 2019-nCov IgG/IgM from the company All Test Biotech., Hangzhou, China 310018), according to the manufacturer’s specifications.

### 2.4. Statistical Analysis

Quantitative variables are expressed as medians (ranges), and qualitative characteristics are described as frequencies (%). The chi-square test (or Fisher’s exact test) was used for comparison proportions between groups. Comparisons of quantitative variables between outpatients with sufficient vitamin D and insufficient vitamin D serum levels, as well as comparisons of outpatients with and without supplementation, were performed using the Mann-Whitney U test. Spearman’s test identified the correlations between vitamin D serum levels and clinical variables. The comparison of vitamin D serum levels in patients with supplementation and those without supplementation was performed using the Wilcoxon Rank-Sum Test. To identify factors associated with a high number of symptoms at baseline, we performed a logistic regressions analysis. We used R version 4.0.3 to perform the statistical analyses and ggplot2 package for graphics. A *p*-value ≤0.05 was considered statistically significant.

## 3. Results

### 3.1. Demographic and Clinical Features of Vitamin D Supplemented and Non-Supplemented COVID-19 Outpatients at Baseline

Demographic features and the pharmacological treatment of the 42 COVID-19 outpatients are described in Table 1. The median age was 43 years and the minimum and maximum ages were 20 and 74, respectively. Regarding sex, 22 females (52.4%) and 20 males (47.6%) were included. Regarding the comorbidities, 16.7% had hypertension, 4.8% diabetes, and 2.4% asthma. Only four outpatients (9.5%) were smokers; however, all subjects reported that they stopped smoking at the onset of symptoms.

The total of outpatients showed median ferritin levels of 129.5 ng/mL (6.62–842) and 236.0 ng/mL of serum transferrin (171.0–376). D-dimer median levels were 286.1 ng/mL (100–2825.6). Regarding vitamin D, it was observed that the levels of total vitamin D in serum corresponded to 22.4 ng/mL, with minimum and maximum values of 12.1 ng/mL and 45.9 ng/mL, respectively. Among all investigated patients, only eight out of the whole population presented levels that correspond to vitamin D sufficiency (≥30 ng/mL).

Most of the enrolled outpatients (71.4%) were following some treatment associated with COVID-19; among these, the most common scheme consisted of analgesics (52.4%), antipyretics (40.5%), anticoagulant (11.9%), antibiotics (19%), antihistamine (14.3%), beta-blockers (4.8%), antiparasitic (9.5%), and flu drugs (14.3). Notably, only four (9.5%) of the total included outpatients were treated with corticosteroids at the inclusion time. D-dimer and ferritin values were also similar in both groups; however, these associations should be interpreted with caution because the *p*-value was equal to 0.05.

The comparison of demographic and clinical features between groups of supplemented and non-supplemented outpatients at baseline showed that both groups were similar in age, comorbidities, number of symptoms, and in the use of different pharmacological treatments. D-dimer and ferritin values were also similar in both groups (*p* ≥ 0.05).

Overall, 83% of the included outpatients provided information regarding their blood type: the highest frequency corresponded to 0 + (37.1%), followed by A + (28.6%), B + (20%), 0 − (11.4%), while the lowest frequency was observed for AB + (2.9%) (data not shown in tables).

### 3.2. Association between Vitamin D Serum Levels and Clinical and Laboratory Variables at Baseline

**Figure 1:**
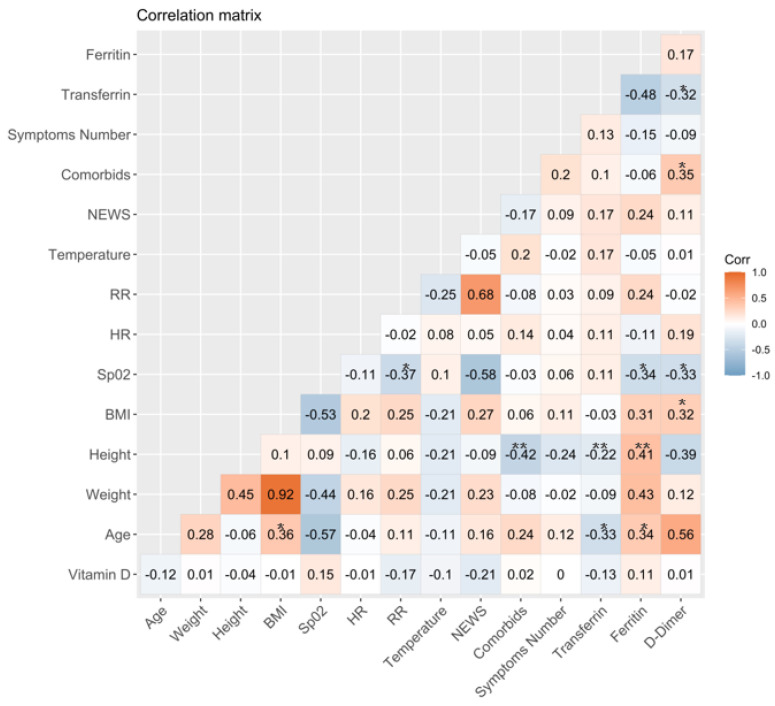
Correlations between clinical and laboratory features of outpatients. Blue colors show negative correlations and orange shows positive correlations (significant correlations are highlighted with a star * *p* ≤ 0.05 or two stars ** *p* ≤ 0.01). NEWS = National Early Warning Score, RR = Respiratory Rate, HR = Heart Rate, BMI = Body Mass Index, SpO2 = Oxygen Saturation.

Figure 1 shows correlations between clinical and laboratory features of outpatients at baseline. Regarding the association between COVID-19 and bad prognosis, biomarkers, such as transferrin, ferritin, and D-dimer, were observed, and serum levels of ferritin correlated positively with age (rs = 0.34) and height (rs = 0.41). Conversely, a negative association was observed between ferritin and oxygen saturation (SpO2) (rs = −0.34). Transferrin serum levels negatively correlated with D-dimer serum levels (rs = −0.32), as well as with age (rs = −0.33) and height (rs = −0.22). Additionally, D-dimer positively correlated with parameters, such as the presence of comorbidities (rs = 0.35) and body mass index (BMI) values (rs = 0.32). Negative correlations were also observed between D-dimer and SpO2 (rs = −0.33), between the height and presence of comorbidities (rs = −0.42), as well as SpO2 and the respiratory rate (RR) (rs = −0.37). Finally, it was also observed that age and BMI were positively associated (rs = 0.36).

### 3.3. Comparison between Laboratory Parameters and Clinical Features of Outpatients with or without Sufficient Levels of Total Vitamin D

Evaluating the baseline concentrations in serum of total vitamin D, it was observed that only eight (19.0%) outpatients had vitamin D levels corresponding to sufficiency (≥30 ng/mL). In contrast, the rest of the outpatients (*n* = 34) had levels < 30 ng/mL, which were considered insufficient (Table 2). Subsequently, when stratifying by vitamin D status, it was observed that all outpatients with vitamin D insufficiency had more than one symptom associated with the disease, while only 75% of outpatients with vitamin D sufficiency presented symptoms associated with COVID-19; this difference was statistically significant (*p* = 0.03). It was also observed that outpatients with vitamin D insufficiency presented a higher frequency of more than one COVID-19 symptom than those with a sufficiency of vitamin D (*p* = 0.03). No significant association was observed with the rest of the parameters evaluated (Table 2).

### 3.4. Effect of Vitamin D3 Supplementation in Total Serum Levels of Vitamin D

**Figure 2:**
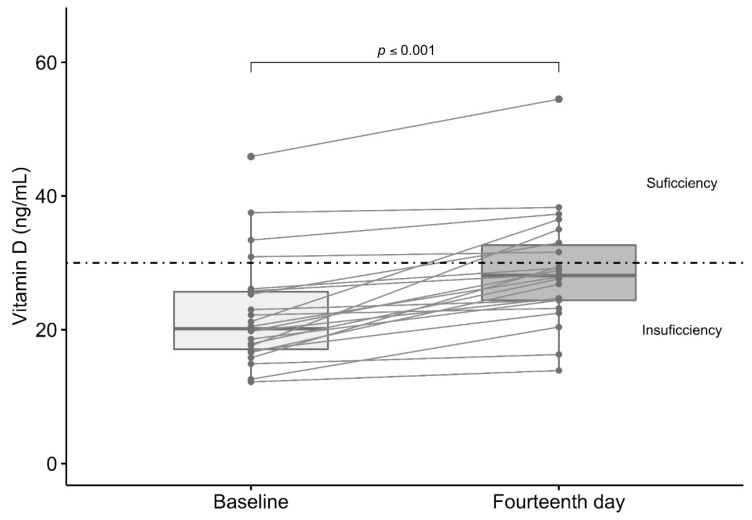
Effect of vitamin D3 supplementation in total serum levels of vitamin D. The light gray color bar shows baseline serum total vitamin D levels in COVID-19 outpatients (*n* = 20) and the dark gray color bar shows total vitamin D levels in these same patients on the fourteenth day after supplementation with 10,000 IU daily of vitamin D3. Comparisons among groups were made using the Wilcoxon Rank-Sum Test.

The intervention group had median levels of total vitamin D of 20.2 ng/mL (12.2–45.9) before vitamin D3 supplementation (10,000 IU daily) for 14 days (average duration of COVID-19 pathogenesis). The administered supplementation was sufficient to increase total vitamin D serum levels significantly on day 14 (28.2 ng/mL (13.9–54.5)) (Figure 2). The proportion of patients with sufficiency vitamin D serum levels (≥30 ng/mL) increased (*p* = 0.04) within 14 days of follow-up (*n* = 7 (31.2%)), with respect to the baseline (*n* = 4 (18.2%)).

### 3.5. Comparison between Symptoms, Treatment, and Viral Load in Supplemented and Non-Supplemented Outpatients Study Groups

The first follow-up of supplemented and non-supplemented outpatients was 7 days after patients had received their positive diagnosis. Outpatients were asked about the number of symptoms that they still presented, as well as the treatment they were taking. Another PCR test was performed to evaluate if they were still positive for SARS-CoV-2 (Table 3). Regarding symptomatology, it was observed that, in general, the supplemented group had fewer symptoms, with differences in those who had more than three symptoms (20% in the group of non-supplemented outpatients and none in the group of supplemented outpatients) (*p* = 0.04). The percentage of positive patients with the PCR test was similar in both groups (*p* > 0.05).

The second follow-up was carried out on the fourteenth day. Regarding symptoms, the same results were observed as on day 7, with a greater number of patients with more than three symptoms in the non-supplemented group compared to the supplemented group (*p* = 0.04). Most outpatients had a negative PCR test; only one outpatient in the non-supplemented group was still positive for SARS-CoV-2 (*p* = 0.47). As for the rest of the variables, no significant differences were observed. The complete comparison between supplemented and non-supplemented outpatients on days 7 and 14 is shown in Table 3.

Additional information about symptoms in supplemented and non-supplemented outpatients at baseline and follow-up at days 7 and 14 is presented as Supplementary Material (Supplementary Table S1).

### 3.6. COVID-19 Outpatients Seropositivity Rate on the Seventh Day of Follow-Up

**Figure 3:**
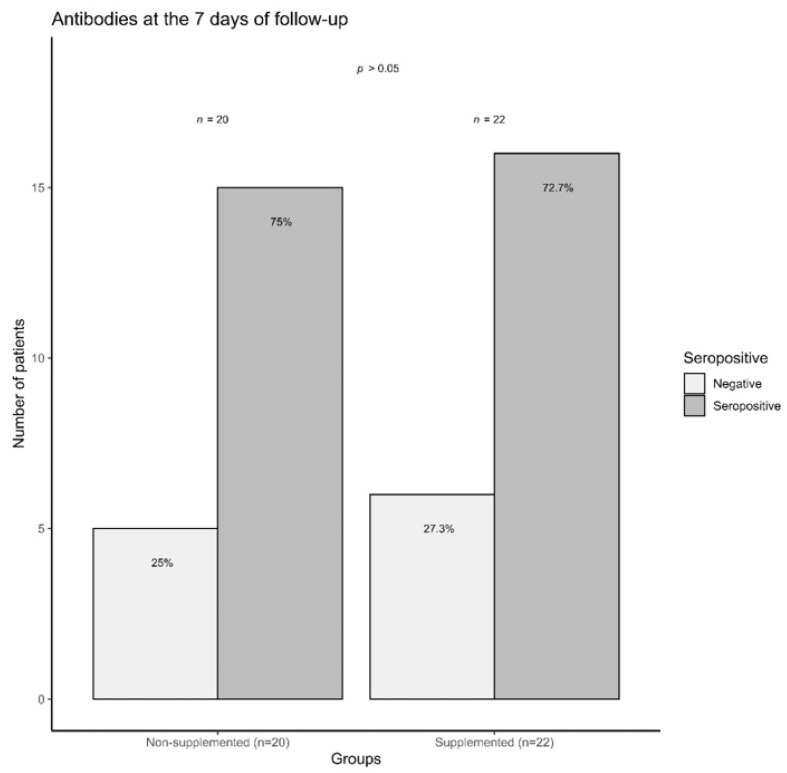
COVID-19 outpatients seropositivity rate (IgM and/or IgG) at day seven. The graph shows the percentage of seropositive (dark gray color) and seronegative (light gray color) outpatients, supplemented and not supplemented with vitamin D3 on day seven. Results obtained by a lateral flow immunoassay method (rapid qualitative test Certum 2019-nCov IgG/IgM). n supplemented = 22, *n* non-supplemented = 20.

Finally, on the seventh day of follow-up, the percentage of seropositivity was evaluated between the intervention and control groups. No differences were observed between supplemented and non-supplemented patients concerning IgM or IgG frequency (seropositive). Additionally, the number of seronegative patients was similar in both groups (*p* > 0.05) (Figure 3).

## 4. Discussion

The outbreak and rapid spread of SARS-CoV-2 are health threats with unprecedented consequences throughout the world. According to several reports, during diagnosis and treatment, changes in biochemical, hematological, and immunological features have been identified as markers of COVID-19 and could predict the worsening of the condition [28,29,30]. In the present work, an analysis of the status of D-dimer, transferrin, and ferritin was performed, which are some of the most commonly used inflammatory markers for monitoring COVID-19 patients.

D-dimer is a biomarker of fibrin formation and degradation, which can be measured in whole blood or plasma [31]. Since the association of coagulopathy with COVID-19 is now widely reported, several studies have established that patients with severe disease were more likely to exhibit dysregulated coagulation function and a significantly higher D dimer level [30,31]. Low levels of D-dimers are detectable in healthy individuals, as small amounts of fibrinogen are converted to fibrin and physiologically normal levels are considered as <500 ng/mL [32]. In this regard, in the present work, median levels of D-dimer in patients were among normal values (306.7 ng/mL).

It is also known that the innate immune response that is triggered in response to COVID-19 infection can restrict the availability of iron to deprive the pathogen of it, a mechanism that would also lead to anemia. Anemia, in turn, reduces the oxygen supply to the tissue and, therefore, can play an important role in the development of multi-organ failure [33]. On the other hand, ferritin is supposed to be a cellular means of storing iron, not transporting it, yet serum ferritin levels are widely measured as indicators of iron status [34], while transferrin is a liver-derived protein able to bind up to two iron atoms in ferric form. Iron-laden-transferrin delivers the metal to most cells upon binding to the transferrin receptor [35]. Both ferritin and transferrin serum levels can be raised significantly in response to inflammation and/or various diseases [34].

Increased ferritin and transferrin levels could indicate a strong inflammatory reaction in COVID-19 or is related to viral entry into the human body and its impact on iron metabolism [33]. In this study, the median levels observed for both parameters are within the concentrations considered normal for healthy individuals (30–300 ng/mL for ferritin and 200–400 mg/dL for transferrin). These results may be due to the fact that the evaluated patients were asymptomatic or with a mild phenotype of the disease, while the previously described associations with D-dimer, transferrin, and ferritin were made in groups of patients with a severe COVID-19 phenotype.

Interestingly, when correlating these biomarkers with different clinical parameters of COVID-19, it was observed that serum levels of ferritin correlated negatively with SpO_2_. These results agree with those reported by Lee et al., 2020, where it was found that high levels of ferritin were associated with decreased lung function in healthy Korean men [36]. According to reports, ferritin can be elevated in oxidative stress and inflammation irrespective of iron status and can contribute to various clinical diseases, especially pulmonary and cardio-metabolic diseases. This suggests that decreased lung function could be associated with elevated serum ferritin levels in pathological conditions.

Meanwhile, D-dimer positively correlated with parameters, such as the presence of comorbidities and BMI values. Regarding the positive association between D-dimer and BMI values, these results agree with a previous study that observed high D-dimer values in obese patients. These associations are explained by the possible contribution of abdominal adiposity to intravascular coagulation and subsequent atherothrombosis [37].

Negative correlations were also observed between D-dimer and SpO_2_. This negative association has been previously described in the work of Shitrit et al. (2005), where it is suggested that the hypoxia is capable of triggering a process of pulmonary vasoconstriction. The hypoxemia state may lead to endothelial damage and abnormal fibrinolysis, which would be reflected in D-dimer levels [38].

In previous clinical trials, it has been reported that levels of inflammatory markers and the decrease in viral load of SARS-CoV-2 are related to supplementation with high doses of vitamin D [39].

The immunomodulatory effects of vitamin D are known to be beneficial in viral infections. Recent clinical trials reported that vitamin D supplementation could reduce the incidence of acute respiratory infection and the severity of respiratory tract diseases in adults and children [40]. Low levels of vitamin D are also associated with chronic diseases, such as ARDS [41], breast cancer [42], and asthma [43]. In this regard, in the Mexican population, Bedolla-Barajas et al. (2017) showed that, in a total of 135 patients with allergic asthma, the prevalence of vitamin D insufficiency and deficiency was 25.2% and 71.1%, respectively [44]. For some autoimmune conditions, vitamin D deficiency has also been related to high levels of pro-inflammatory cytokines, such as TNF-α and IFN-γ [45].

Alterations in vitamin D concentrations, either deficiency (serum levels < 20 ng/mL) or insufficiency (serum levels 20–29.9 ng/mL), represent a global health problem [27]. According to the committee of the Institute of Medicine, people are at risk of the condition called “hypovitaminosis D” at serum concentrations of 25(OH)D < 30 nmol/L (<12 ng/mL) [46]. It is also well known that differences in geographic location, skin color, and type of diet play an important role in the body disposition and metabolism of vitamin D [13,14,24]. In this sense, it has also been observed in the Mexican population that vitamin D deficiency is a common condition [47].

In the present study, baseline total vitamin D serum levels were measured in COVID-19 outpatients. We observed that only 19% of COVID-19 outpatients had vitamin D levels corresponding to sufficiency (≥30 ng/mL); the rest of the patients (81%) had levels < 30 ng/mL, which are considered as insufficient. These results agree with those reported by Pinzon (2020), which showed vitamin D deficiency in 90% (vitamin D levels < 20 ng/mL) of COVID-19 patients from Indonesia, and the remaining 10%, presented insufficiency (vitamin D levels < 30 ng/mL) [48]. Similar results were observed by Yılmaz, K. et al. in hospitalized COVID-19 pediatric patients, where statistically lower vitamin D levels were observed compared to healthy individuals (*p* < 0.001) [19].

As described before, supplementation with high doses of vitamin D is necessary to achieve concentrations above 30 ng/mL (levels established as “sufficiency”) in individuals with vitamin D deficit. According to previous studies, a dose of 10,000 IU daily for a few weeks is adequate to increase serum levels of vitamin D in patients with low circulating levels (below 50 nmol/L). It has also been reported that doses of up to 15,000 IU daily are safe and effective to rapidly increase vitamin D serum concentrations, followed by a maintenance dose of up to 5000 IU daily [5,25].

Due to the general vitamin D deficiency in the Mexican population, we aimed to find a vitamin D dose to increase the serum levels in a short period. In this regard, a vitamin D supplementation equivalent to 10,000 IU per day was administered for 14 days in the intervention group. We observed that this dose was sufficient to significantly increase levels in 14 days without any side effects. These results could serve as a prelude to establish an efficient and safe vitamin D3 supplementation dose to increase serum levels in individuals from Mexico.

Furthermore, when we stratified the patients by vitamin D status, it was observed that most of the patients presented levels corresponding to vitamin D insufficiency, and these results represent a reflection of the general state of hypovitaminosis in the Mexican population. In addition to this, it was observed that those with insufficiency had at least one symptom associated with the disease, while only 75% of patients with vitamin D sufficiency presented symptoms associated with COVID-19. The number of symptoms was also higher in patients with vitamin D insufficiency; 91.2% of patients with vitamin D insufficiency presented more than one symptom, while only 62.5% of those with vitamin D sufficiency presented the same number symptoms. A similar association was observed on the seventh and fourteenth day of follow-up, where the supplemented group presented fewer symptoms, with respect to those who had more than three symptoms (20% in the group of non-supplemented outpatients and none in the group of supplemented outpatients).

These results are similar to those reported by Yılmaz, K. and Şen, V. (2020) in pediatric patients with vitamin D deficiency and insufficiency, where it was observed that the specific symptom of fever (34.5%) was significantly higher when compared to the group with normal vitamin D levels [19]. Similarly, the report by Nowaczewska et al. established that low levels of vitamin D have been directly associated with headaches and chronic musculoskeletal pain [49].

On the other hand, Ye et al. (2020) demonstrated that, in patients with COVID-19 treated at the Yongwu Hospital of The People’s Hospital of Guangxi Zhuang Autonomous Region, all potential risk factors as independent variables (including age, sex, renal failure, diabetes, and hypertension) indicate a statistically significant association between vitamin D deficiency and severe/critical disease (OR, 15.18; 95% CI, 1.23–187.45) [50].

The above can be explained by vitamin D effects on most immune system cells, such as macrophages, B and T lymphocytes, neutrophils, and dendritic cells [13]. Vitamin D can inhibit the production of pro-inflammatory cytokines and favor the production of anti-inflammatory cytokines [15,16]. Vitamin D can also inhibit the adaptive immune system and promote the innate immune system that balances the immune response and provides a general anti-inflammatory response [51]. Likewise, vitamin D has been shown to regulate the renin-angiotensin system and ACE2 expression (host receptor for SARS CoV-2) in animal models [52]. Therefore, the possibility that vitamin D deficiency causally increases disease severity amongst some patients infected with SARS-CoV-2 is biologically plausible, given the distinct immunophenotype and other biochemical aberrations that characterize both severe COVID-19 and vitamin D deficiency [24].

In general, the immunomodulatory effects of vitamin D appear to be related to the development of symptoms in outpatients with COVID-19. Based on our results, we suggest that appropriate levels of vitamin D prior to COVID-19 infection could help to counteract severe symptoms of the disease. Therefore, vitamin D supplementation would have great benefits in the Mexican population due to the high rate of insufficiency observed in this study and results reported by others [44,47,53].

Our study also shows that a vitamin D3 dose of 10,000 IU daily for 14 days was sufficient to raise vitamin D concentrations. To date, reports of adequate concentrations of vitamin D supplementation in the Mexican population are scarce; in that sense, it is to our knowledge that this is the first study that reports baseline vitamin D status in COVID-19 outpatients from Mexico.

Among the weaknesses of this study, it is important to mention that, although baseline serum vitamin D levels were determined in both groups, they were not reported on day 14 in the group of control subjects; these were determined in the group of outpatients supplemented to evaluate the effect of the intervention. The foregoing, in addition to the fact that the study was not designed as double blind, represent the same weaknesses as those considered for the subsequent work that we intend to carry out in this same population. In the same way, another important area of opportunity of this study in the future will be to determine the serum levels of pro- and anti-inflammatory cytokines, which, as reported, are fundamental in the pathogenesis of the disease, such as IL-6, IL-8, and TNF-α [4,5]. Finally, in subsequent studies, further studies will be important to increase the sample size of both study groups and consider the aspects above to evaluate the effect of vitamin D3 supplementation in patients with COVID-19 in the Mexican population on a large scale.

## Figures and Tables

**Table 1:** Demographic and treatment features of positive COVID-19 outpatients at baseline.

Variable	*n* = 42	Supplemented Outpatients *n* = 22	Non-Supplemented Outpatients *n* = 20	*p*-Value
Age (years) ^a^	43.0 (20–74)	44.0 (20.0–71.0)	43.0 (21.0–78.0)	0.66
Females ^b^	22 (52.3)	7 (31.8)	6 (30.0)	1.00
BMI (kg/m^2^) ^a^	25.5 (18.1–41.2)	25.4 (19.7–41.2)	26.3 (18.1–35.0)	0.95
Comorbidities				
- Arterial Hypertension ^b^	7 (16.7)	4 (18.2)	3 (15.0)	0.55
- Smoke ^b^	4 (9.5)	2 (9.1)	2 (10.0)	1.00
- Diabetes mellitus ^b^	2 (4.8)	0 (0.0)	2 (10.0)	0.22
- Asthma ^b^	1 (2.4)	1 (2.4)	0 (0.0)	1.00
Treatment	30 (71.4)	15 (68.2)	15 (75.0)	0.88
- Analgesic ^b^	22 (52.4)	12 (54.5)	10 (50.0)	1.00
- Antipyretic ^b^	17 (40.5)	7 (31.8)	10 (50.0)	0.37
- Antibiotic ^b^	8 (19.0)	2 (9.1)	6 (30.0)	0.12
- Antihistamine ^b^	6 (14.3)	3 (13.6)	3 (15.0)	1.00
- Anticoagulant ^b^	5 (11.9)	1 (4.5)	4 (20.0)	0.17
- Other drugs ^b^	10 (23.8)	5 (22.7)	5 (25.0)	1.00
Laboratory parameters				
- Ferritin (ng/mL) ^a^	129.5 (6.62–842.0)	72.8 (8.6–419.0)	153.0 (6.62–842)	0.05
- D-dimer (ng/mL) ^a^	286.1 (100–2825.6)	306.7 (100–2825.6)	263.6 (186.0–1038.5)	0.89
- Transferrin (ng/mL) ^a^	236.0 (171.0–376.0)	254.0 (193.0–376.0)	226.0 (171.0–297)	**0.03**
Total vitamin D (ng/mL) ^a^	22.4 (12.1–45.9)	20.2 (12.2–45.9)	23.4 (12.1–45.6)	0.06
Sufficient vitamin D ^b^	8 (19.0)	4 (18.2)	4 (20.0)	1.00

^a^ = Quantitative variables are expressed as the median and range (minimum–maximum). ^b^ = Qualitative variables are expressed as frequency and percentages. *p*-values with statistical significance are highlighted in bold. Ct = Cycle threshold of RT-PCR. Mann-Whitney-U test was performed for comparison of quantitative variables *p* ≤ 0.05. Chi square test of the Fisher exact test was performed for comparison of qualitative variables *p* ≤ 0.05. BMI = body mass index.

**Table 2:** Comparison between outpatients with sufficient and insufficient levels of total vitamin D at baseline.

Variable	Outpatients with Insufficient Levels of Vitamin D *n* = 34	Outpatients with Sufficient Levels of Vitamin D *n* = 8	*p*-Values
Age (years) ^a^	45 (20–74)	38.5 (36–64)	0.42
Comorbid ^b^	11 (32.4)	2 (25.0)	0.70
BMI (kg/m^2^) ^a^	25.5 (18.1–39.2)	25.9 (19.6–41.2)	0.75
Symptoms ^b^	34 (100.0)	6 (75.0)	**0.03**
>1 symptom ^b^	31 (91.2)	5 (62.5)	**0.03**
>2 symptoms ^b^	27 (79.4)	5 (62.5)	0.37
>3 symptoms ^b^	23 (67.6)	4 (50.0)	0.42
>4 more symptoms ^b^	18 (52.9)	4 (50.0)	0.59
Number of symptoms ^a^	6 (0–11)	5 (0–10)	0.36
Treatment ^b^	24 (72.7)	6 (75.0)	0.89
Laboratory parameters			
- Transferrin (mg/dL) ^b^	237.0 (178.0–376.0)	234 (171.0–289.0)	0.72
- Ferritin (ng/mL) ^b^	119–5 (6.6–842.0)	186.5 (70.5-453)	0.18
- D-dimer (ng/mL) ^b^	278.4 (100.0–1239.9)	3310.4 (128.9–2825.6)	0.56

^a^ = Quantitative variables are expressed as the median and range (minimum—maximum). ^b^ = Qualitative variables are expressed as frequency and percentages. *p*-values with statistical significance are highlighted in bold. The Mann-Whitney-U test was performed for comparison of quantitative variables *p* ≤ 0.05. Chi square test of the Fisher exact test was performed for comparison of qualitative variables *p* ≤ 0.05. BMI = body mass index.

**Table 3:** Comparison of symptoms, treatment, and viral load in supplemented and non-supplemented outpatient study groups at baseline and follow-up at days 7 and 14.

	Baseline				7 Days			14 Days	
Variable	Supplemented Outpatients *n* = 22	Non-supplemented Outpatients *n* = 20	*p*-Value	Supplemented Outpatients *n* = 22	Non-supplemented Outpatients *n* = 20	*p*-Value	Supplemented Outpatients *n* = 22	Non-Supplemented Outpatients *n* = 20	*p*-Value
Presence of symptoms	21 (95.5)	19 (95.0)	1.00	13 (59.1)	9 (45.0)	0.53	14 (63.6)	8 (40.0)	0.22
>1 symptom ^a^	18 (81.8)	18 (90.0)	0.66	5 (22.7)	6 (30.0)	0.43	6 (27.3)	6 (30.0)	1.00
>2 symptoms ^a^	17 (77.3)	15 (75.0)	1.00	2 (9.1)	4 (20.0)	0.28	4 (18.2)	4 (20.0)	0.59
>3 symptoms ^a^	14 (63.6)	13 (65.0)	1.00	0 (0.0)	4 (20.0)	**0.04**	0 (0.0)	4 (20.0)	**0.04**
NEWS score ^b^	4 (0–9)	3 (0–7)	0.14	---	----	---	---	----	---
Treatment ^a^	15 (68.2)	15 (75.0)	0.88	7 (31.8)	8 (40.0)	0.81	4 (18.2)	4 (20.0)	0.88
Analgesic ^a^	12 (54.5)	10 (50.0)	1.00	2 (9.1)	3 (15.0)	0.57	2 (9.1)	3 (15.0)	0.65
Antipyretic ^a^	7 (31.8)	10 (50.0)	0.35	4 (18.2)	6 (30.0)	0.53	1 (4.5)	3 (15.0)	0.27
Antibiotic ^a^	2 (9.1)	6 (30.0)	0.12	0 (0.0)	2 (10.0)	0.12	1 (4.5)	0 (0.0)	1.00
Antihistamine ^a^	3 (13.6)	3 (15.0)	1.00	1 (4.5)	0 (0.0)	1.00	0 (0.0)	0 (0.0)	---
Anticoagulant ^a^	1 (4.5)	4 (20.0)	0.17	0 (0.0)	1 (5.0)	1.00	0 (0.0)	0 (0.0)	---
Other drugs ^a^	5 (22.7)	5 (25.0)	1.00	4 (18.8)	3 (15.0)	1.00	0 (0.0)	0 (0.0)	---
Positive RT-PCR test ^a^	22 (100.0)	20 (100.0)	---	12 (60.0)	12 (54.5)	0.97	1 (5.0)	0 (0.0)	0.47

^a^ = Quantitative variables are expressed as medians and range (minimum–maximum). ^b^ = Qualitative variables are expressed as frequency and percentages. *p*-values with statistical significance are highlighted in bold. Chi square test of the Fisher exact test was performed for comparison of qualitative variables *p* ≤ 0.05. Other drugs included: antiparasitic, Corticoids, and beta blockers. NEWS = National Early Warning Score, RT-PCR = Retrotranscription polymerase chain reaction. NEWS were evaluated at base-line. *p*-values with statistical significance are highlighted in bold.

## Data Availability

The data presented in this study are not publicly available.

## Supplementary Materials

The following are available online at https://www.mdpi.com/article/10.3390/jcm10112378/s1, Table S1: Comparison of symptoms in supplemented and non-supplemented outpatients study groups at baseline and follow-up at days 7 and 14.
