# Supplementary material for: Vitamin D Levels in COVID-19 Outpatients from Western Mexico: Clinical Correlation and Effect of Its Supplementation
Source: J Clin Med. 2021 May 28;10(11):2378. doi: 10.3390/jcm10112378 (PMC8198869; doi:10.3390/jcm10112378)
Supplement: Supplementary file 1 [file jcm-10-02378-s001.zip › jcm-1165567-supplementary.pdf]

Supplementary Table S1. Comparison of symptoms in supplemented and non-supplemented outpatients study groups at baseline and follow-up at days 7 and 14

Variable	Base-line			7 days			14 days		
	Supplemented outpatients n=22	Non-supplemented outpatients n=20	p-value	Supplemented outpatients n=22	Non-supplemented outpatients n=20	p-value	Supplemented outpatients n=22	Non-supplemented outpatients n=20	p-value
Fever [a]	9 (40.9)	8 (40.0)	0.95	0 (0.0)	2 (10.0)	0.22	0 (0.0)	2 (10.0)	0.22
Headache [a]	14 (64.3)	13 (65.0)	0.93	4 (18.2)	3 (15.0)	1.00	4 (18.2)	3 (15.0)	1.00
Loss of smell [a]	10 (45.5)	11 (55.0)	0.54	3 (13.6)	1 (50.0)	0.61	3 (13.6)	1 (5.0)	0.61
Dry cough [a]	13 (59.1)	15(75.0)	0.27	4 (18.2)	4(20.0)	1.00	4 (18.2)	4 (20.0)	1.00
Sore throat [a]	6 (27.3)	7 (35.0)	0.58	1 (4.5)	0 (0.0)	1.00	1 (4.5)	0 (0.0)	1.00
Ageusia [a]	10 (45.5)	12 (60.0)	0.34	3 (13.6)	4 (20.0)	0.69	3 (13.6)	4 (20.0)	0.69
Runny nose [a]	3 (13.6)	5 (25.0)	0.44	0 (0.0)	1 (5.0)	0.48	0 (0.0)	0 (0.0)	---
Nausea or [a] Vomiting [a]	3 (13.6)	6 (30.0)	0.27	0 (0.0)	3 (15.0)	0.09	0 (0.0)	3 (15.0)	0.09
Tiredness [a]	8 (36.4)	12 (60.0)	0.13	4 (18.2)	4 (20.0)	1.00	5 (22.7)	4 (20.0)	1.00
Diarrhoea [a]	5 (22.7)	2 (25.0)	1.00	0 (0.0)	1 (5.0)	0.48	0 (0.0)	0 (0.0)	---
Myalgia [a]	12 (54.5)	20 (60.0)	0.72	1 (4.5)	2 (10.0)	0.60	1 (4.5)	2 (10.0)	0.60
Arthralgia [a]	8 (36.4)	12 (60.0)	0.13	0 (0.0)	0 (0.0)	---	0 (0.0)	0 (0.0)	---
Shortness of breath [a]	2 (9.1)	0 (0.0)	0.48	0 (0.0)	3 (15.0)	0.09	0 (0.0)	0 (0.0)	---

a= Qualitative variables are expressed as frequency and percentages. *p*-values with statistical significance are highlighted in bold. Chi square test o Fisher exact test was performed for comparison of qualitative variables $p \leq 0.05$.
